# PSMB8 stratifies therapy response in eosinophilic esophagitis

**DOI:** 10.1093/cei/uxag012

**Published:** 2026-04-07

**Authors:** Xinyi Wei, Sabrina Degen, Theresa Hironimus, Tobias Rechenauer, Katharina Yankouskaya, Aline Rückel, Margit Schmid, Daniel Rieger, Christoph Ehrsam, Adrian P Regensburger, Alexander Schnell, Anja Rabe, Anjona Schmidt-Choudhury, Andrea Tannapfel, Jan De Laffolie, Stefan Schumann, Tobias Schwerd, Hannes Hoelz, Ida Allabauer, Pooja Gupta, Wolfgang Krebs, Arndt Hartmann, Joachim Woelfle, Ralf Rieker, Andre Hoerning

**Affiliations:** Pediatric Gastroenterology, Hepatology and Endoscopy, Department of Pediatrics and Adolescent Medicine, University Hospital Erlangen, Friedrich-Alexander-University Erlangen-Nürnberg, Erlangen, Germany; Department of Obstetrics and Gynecology, The Third Affiliated Hospital of Zhengzhou University, Zhengzhou, China; Pediatric Gastroenterology, Hepatology and Endoscopy, Department of Pediatrics and Adolescent Medicine, University Hospital Erlangen, Friedrich-Alexander-University Erlangen-Nürnberg, Erlangen, Germany; Pediatric Gastroenterology, Hepatology and Endoscopy, Department of Pediatrics and Adolescent Medicine, University Hospital Erlangen, Friedrich-Alexander-University Erlangen-Nürnberg, Erlangen, Germany; Pediatric Gastroenterology and Hepatology, Klinikum Dritter Orden, Munich, Germany; Pediatric Gastroenterology, Hepatology and Endoscopy, Department of Pediatrics and Adolescent Medicine, University Hospital Erlangen, Friedrich-Alexander-University Erlangen-Nürnberg, Erlangen, Germany; Pediatric Gastroenterology, Hepatology and Endoscopy, Department of Pediatrics and Adolescent Medicine, University Hospital Erlangen, Friedrich-Alexander-University Erlangen-Nürnberg, Erlangen, Germany; Pediatric Gastroenterology, Hepatology and Endoscopy, Department of Pediatrics and Adolescent Medicine, University Hospital Erlangen, Friedrich-Alexander-University Erlangen-Nürnberg, Erlangen, Germany; Pediatric Gastroenterology, Hepatology and Endoscopy, Department of Pediatrics and Adolescent Medicine, University Hospital Erlangen, Friedrich-Alexander-University Erlangen-Nürnberg, Erlangen, Germany; Department of Pediatrics and Adolescent Medicine, Helios Hospital Meiningen, Meiningen, Germany; Pediatric Gastroenterology, Hepatology and Endoscopy, Department of Pediatrics and Adolescent Medicine, University Hospital Erlangen, Friedrich-Alexander-University Erlangen-Nürnberg, Erlangen, Germany; Pediatric Gastroenterology, Hepatology and Endoscopy, Department of Pediatrics and Adolescent Medicine, University Hospital Erlangen, Friedrich-Alexander-University Erlangen-Nürnberg, Erlangen, Germany; Pediatric Gastroenterology, Helios Klinikum Erfurt, Erfurt, Germany; Department for Pediatric Gastroenterology and Hepatology, Katholisches Klinikum Bochum, Ruhr University Bochum, Bochum, Germany; Institut für Pathologie, Ruhr University Bochum, Bochum, Germany; Department of Pediatrics, Justus-Liebig-University Giessen, Giessen, Germany; Department of Pediatrics, Justus-Liebig-University Giessen, Giessen, Germany; Department of Pediatrics, Dr. von Hauner Children's Hospital, University Hospital, LMU Munich, Munich, Germany; Department of Pediatrics, Dr. von Hauner Children's Hospital, University Hospital, LMU Munich, Munich, Germany; Pediatric Gastroenterology, Hepatology and Endoscopy, Department of Pediatrics and Adolescent Medicine, University Hospital Erlangen, Friedrich-Alexander-University Erlangen-Nürnberg, Erlangen, Germany; Core Unit for Bioinformatics, Data Integration and Analysis, Medical Center for Information and Communication Technology, Universitätsklinikum Erlangen, Erlangen, Germany; Core Unit for Bioinformatics, Data Integration and Analysis, Medical Center for Information and Communication Technology, Universitätsklinikum Erlangen, Erlangen, Germany; Department of Pathology, University Hospital Erlangen, Friedrich-Alexander-University Erlangen-Nürnberg, Erlangen, Germany; Pediatric Gastroenterology, Hepatology and Endoscopy, Department of Pediatrics and Adolescent Medicine, University Hospital Erlangen, Friedrich-Alexander-University Erlangen-Nürnberg, Erlangen, Germany; Department of Pathology, University Hospital Erlangen, Friedrich-Alexander-University Erlangen-Nürnberg, Erlangen, Germany; Pediatric Gastroenterology, Hepatology and Endoscopy, Department of Pediatrics and Adolescent Medicine, University Hospital Erlangen, Friedrich-Alexander-University Erlangen-Nürnberg, Erlangen, Germany

**Keywords:** eosinophilic esophagitis, proton pump inhibitor, PSMB8, STAT-6, Eotaxin-3, children

## Abstract

Proton pump inhibitors (PPIs) are an effective first-line treatment for eosinophilic esophagitis (EoE). However, half of the patients are refractory to PPI therapy, and predictive markers for therapy decision are lacking. Thus, this study aimed to investigate the differences in esophageal immunologic transcriptome between PPI-non-responders and PPI-responders and identify molecular biomarkers to guide therapy decisions. Forty-eight pediatric EoE patients were enrolled and classified due to PPI-therapy response. Pre-treatment esophagus biopsy was collected for gene expression analysis, differentially expressed genes (DEGs) between PPI-responders and non-responders were identified, followed by gene enrichment and protein–protein interaction network analyses. Expression of identified hub genes was confirmed by immunohistochemistry in an extended cohort comprising 62 patients. PPI-non-responders and responders exhibit a partially different transcriptomic profile, as 12 DEGs were up-regulated and one down-regulated. These DEGs are closely related to antigen processing and presentation function. PSMB8 was identified as a hub gene differing between these two groups, and immunohistochemistry confirmed significantly increased expression in PPI-non-responders (*P* < 0.0001). Notably, receiver operating characteristic curves curve analysis of PSMB8 reveals it as highly predictive for PPI response (sensitivity/specificity: 0.61/1.00). PPI-non-responding EoE patients exhibited a more profound dysregulation of gene expression. PSMB8 represents a promising esophageal biomarker for predicting therapy response in pediatric EoE.

## Teaser

PPI-non responsive patients exhibit a more pronounced dysregulation of the immunological transcriptome in the esophageal mucosa compared to PPI-responding patients. Activation of antigen processing and presentation pathways may hamper the success of PPI therapy in EOE patients. PSMB8 may serve as predictive biomarkers for PPI therapy to stratify pediatric EoE patients for effective remission-inducing therapy.

## Graphical Abstract

**Graphical Abstract:**
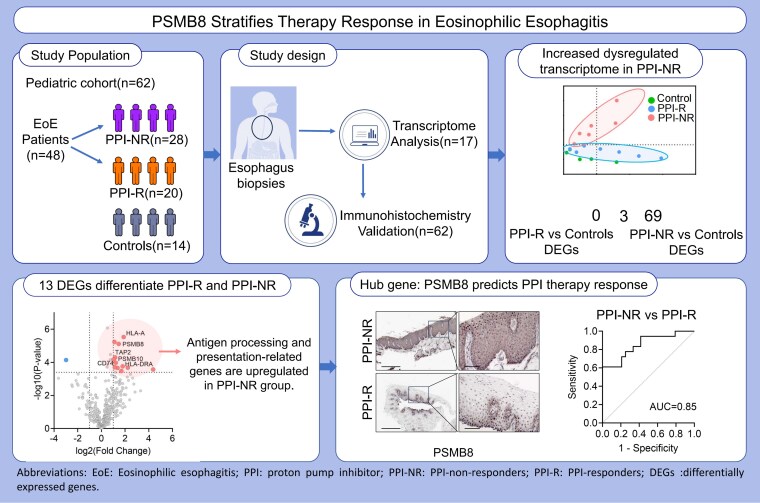

## Introduction

Eosinophilic esophagitis (EoE) is a chronic allergen/immune-mediated type 2 inflammatory disease characterized by esophageal mucosal eosinophilia and esophageal dysfunction [1]. A large proportion of EoE patients achieve clinical and histological remission after high-dose proton pump inhibitor (PPI) treatment [2]. However, accumulating evidence shows that the clinical, endoscopic, and histologic features of PPI-non-responsive and PPI-responsive patients seem to be indistinguishable [3–5].

Studies on PPI response mechanisms suggested both antacid and anti-inflammatory effects in telomerase-immortalized esophageal squamous cell lines from EoE patients. Specifically, PPI inhibits IL-4 and IL-13-induced eotaxin-3 mRNA and protein expression by hampering STAT-6 activity [6–8]. Similar effects were observed in human sinonasal and bronchial epithelial cell lines [9]. However, these findings are primarily from *in vitro* experiments, while the actual mechanisms of PPI responsiveness in EoE patients remain unclear. In this context, reliable predictors of PPI response are unknown, preventing an individualized decision between first-line options such as PPI, elimination or elementary diet, or topical glucocorticosteroids.

Bioinformatic analysis represents an effective tool for interpreting complex gene expression data and disease mechanisms. Comprehensive use of gene expression panels and bioinformatic analysis might help to stratify EoE-specific endo- and phenotypes, and explore the biological mechanisms underlying PPI-responsiveness in EoE patients. Previous transcriptome studies comparing PPI-non-responders (PPI-NR) and PPI-responders (PPI-R) showed very modest differences [10–12], possibly due to the limited gene detection number or small sample sizes. We hypothesized that, despite the similarities, undetected transcriptome differences may still exist. Thus, this study aimed to provide a comprehensive overview of esophageal immunologic transcriptome differences in pediatric PPI-R and PPI-NR patients, to elucidate the underlying mechanisms and identify biomarkers facilitating therapy decisions and monitoring.

## Materials and methods

### Patients and samples

Pediatric patients (≤18 years) who underwent upper gastrointestinal endoscopy and esophagus biopsy from the Clinic of Children and Adolescent Medicine of the Friedrich-Alexander University Erlangen-Nuremberg (FAU) with clinical signs for EoE were prospectively recruited.

According to consensus guidelines [2], EoE was diagnosed in patients presenting with symptoms of esophageal dysfunction and histological evidence of ≥15 eosinophils per high-power field (Eos/HPF) on biopsy. EoE patients treated with first-line PPI-therapy (2 mg/kg/day, divided into two single doses) were enrolled and classified as PPI-R or PPI-NR according to the treatment response. PPI-NR patients were defined as a lack of symptomatic improvement and failure to achieve histological remission after 8 weeks of PPI therapy. PPI-R patients were defined as symptomatic relief and histological remission (<15 Eos/HPF) after PPI therapy. Pediatric subjects with gastrointestinal symptoms undergoing esophagogastroduodenoscopy with biopsy were included in the control group if the esophageal histology was physiological and there was no history of esophageal or allergic diseases.

Exclusion criteria were eosinophilic gastritis and/or enteritis, Crohn’s disease, achalasia, hypereosinophilic syndrome, drug hypersensitivity reactions, infections, connective tissue disorders, and current therapy with PPI, immunosuppressive, or modulatory drugs [2].

**Figure 1:**
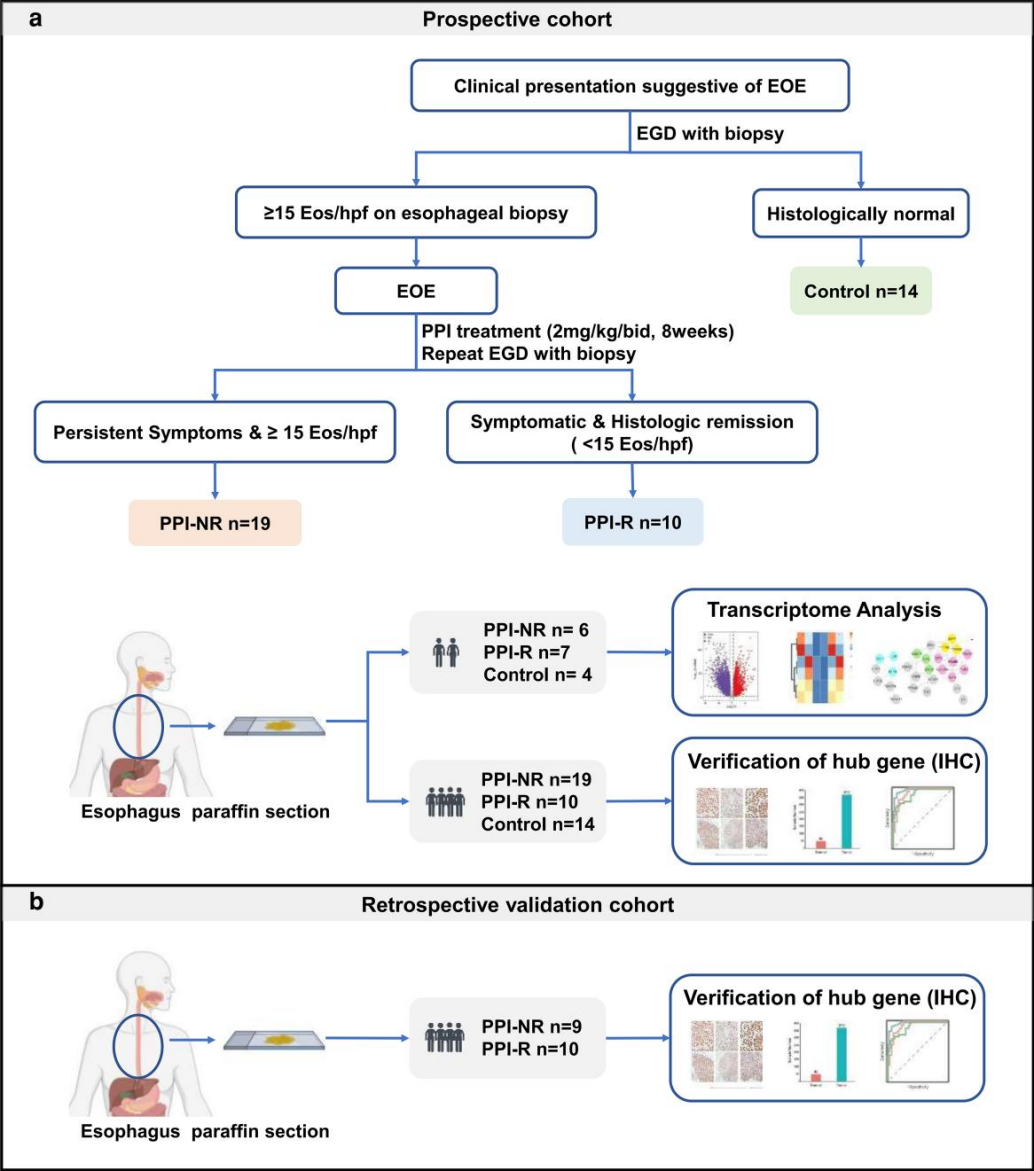
Patient cohort overview. EoE patients treated with PPI-therapy (2 mg/kg per day divided into two single doses) first line were enrolled and classified as PPI-R or PPI-NR according to the treatment response. EGD: esophagogastroduodenoscopy; IHC: immunohistochemistry. Created in BioRender. ›O, _Ã. (2026). https://BioRender.com/a1dus3k.

A total of 62 pediatric subjects were enrolled. Among them, 43 pediatric subjects (19 PPI-NR, 10 PPI-R, and 14 healthy controls) from University Hospital Erlangen were prospectively enrolled. Gene expression profiling was performed on esophageal biopsy samples from six PPI-NR, seven PPI-R, and four control subjects. Immunohistochemistry (IHC) was then conducted on esophageal FFPE samples from all 43 subjects from Erlangen to validate the hub genes identified by transcriptomic analysis (Fig. 1).

Additionally, we retrospectively investigated samples from 19 pediatric EoE patients (9 PPI-NR and 10 PPI-R) from four further German institutions (Northwestern, central, and South Germany) for external validation (Fig. 1).

All subjects included in our study were newly diagnosed and treatment-naïve. Esophageal biopsies were collected from all subjects before initiating PPI therapy, and post-treatment biopsies were continuously collected to assess therapeutic response. Clinical data, including age, gender, symptom, allergic background, endoscopic appearance (EREFS-score) [13], and histology eosinophil count were also collected.

### Ethics statement

The study was approved by the local ethics committee (#317_16B) and registered at DRKS #00014688. Written informed consent was obtained from all participants and/or their legal guardians prior to study inclusion.

### RNA extraction

mRNA from FFPE esophageal specimens was extracted using the RNeasy FFPE Kit (Qiagen, Hilden, Germany). RNA purity and quality were assessed (Thermo Fisher spectrophotometer, Waltham, USA). The A260/A280 ratio was within the range of 1.7–2.3, the A260/A230 ratio within 1.8–2.3, and the RNA total input quality ≥ 100 ng (volumes: 5 µl, concentration: 20–60 ng/µl).

### Differential expression analysis

Expression profiling of 594 immune-related genes in esophageal specimens was performed using the nCounter^®^ Human Immunology v2Panel (XT-CSO-HIM2-12). Raw data were processed and quality controlled using nSolver Analysis SoftwareV4.0 (NanoString Technologies Inc.) and Advanced Analysis Module with R software (R Foundation, Vienna, Austria, V3.3.2). Raw data were normalized using positive control genes (POS_A, POS_B, POS_C, POS_D, POS_E, and POS_F), the housekeeping genes (ABCF1, ALAS1, EEF1G, G6PD, GAPDH, GUSB, HPRT1, OAZ1, POLR1B, POLR2A, PPIA, SDHA, TBP, TUBB, and RPL19), and the geNorm module of the advanced nSolver analysis 4.0 software (MAN-C0019-08). *P*-values were corrected for multiple hypothesis testing using the False Discovery Rate method (Benjamini–Yekutieli) [14]. Log_2_FC (fold change) > 1 and adjusted *P*-values (adj. *P*) < 0.05 were considered as differentially expressed genes (DEGs). The heatmaps were plotted by the Pheatmap V1.0.12 package [15] in the R software statistical analysis platform. Principal component analysis (PCA) was performed by the Scatterplot3d V0.3-42 package in R software. The volcano plot was generated by GraphPad Prism 9.4.0 to display each gene’s −log10 (*P*-value) and Log_2_FC. The common and excluded DEGs among PPI-NR and PPI-R were identified by Online Draw Venn Diagram (http://bioinformatics.psb.ugent.be/webtools/Venn/).

### Functional and pathway enrichment analysis

Gene ontology (GO) and Kyoto Encyclopedia of Genes and Genomes (KEGG) analysis were performed to characterize the biological attributes and pathways of DEGs [16, 17]. GO and KEGG enrichment analyses were produced and visualized through the Database for Annotation, Visualization, and Integrated Discovery (DAVID, V2023q3, last queried on date) [18, 19] and GraphPad Prism 9.4.0. The full gene list from nCounter^®^ Human Immunology v2 Panel was employed as the custom background for conducting enrichment analysis on the DAVID website.

### Construction of protein–protein interaction network and identification of hub genes

Protein–protein interaction network of DEGs was established by STRING V11.5 (http://www.string-db.org/) [20]. The cut-off standard was considered as an interaction score > 0.4 (medium confidence). Next, the protein–protein interaction network of DEGs was visualized by Cytoscape V3.9.1 [21]. The significant gene cluster modules were identified by a Cytoscape plug-in-MCODE (Molecular Complex Detection) V2.0.1 [22]. Subsequently, the Cytohubba V0.1 plugin [23] was employed to study node centrality within the network using multiple algorithms, such as MCC level, to identify the hub genes.

### Immunohistochemistry

Paraffin-embedded esophageal sections were deparaffinized in xylene for 10 min and rehydrated sequentially in 100%, 95%, and 70% ethanol for 6 min each. Antigen retrieval was performed by incubating the samples in citrate buffer (pH = 6.0) for 25 min. Endogenous biotin was blocked with Avidin/Biotin Blocking Kit (Vector, Newark, CA, USA) according to the manufacturer's recommendations, followed by a 20 min blocking step with 10%FCS + 10%goat serum + 80%protein block serum (Dako, Glostrup, Denmark). Tissue sections were incubated with the rabbit anti-human PSMB8 monoclonal antibody (JB54-32, Invitrogen, Waltham, USA) overnight at 4°C. Endogenous peroxidases were blocked with 3% hydrogen peroxide (Roth, Karlsruhe, Germany) for 30 min at room temperature. Subsequently, the slices were incubated with goat anti-rabbit biotinylated antibody (Invitrogen) for 1 h at room temperature. The Avidin-Biotin complex kit (VECTASTAIN, Vector) was utilized for signal detection, with DAB Substrate Kit (Vector) as the chromogen substrate. Sections were counterstained with hematoxylin, dehydrated with graded EtOH and xylene, and finally mounted with organic mounting media (Eukitt, Sigma-Aldrich, St. Louis, MO, USA). IHC slides were imaged using the light microscope (Zeiss AXIO Scope.A1) and recorded with AxioVision Rel.4.8.

Semi-quantitative analyses of IHC TIFF images were performed using the Color Deconvolution plugins V3.0.3 [24] from FIJI V2.9.0 [25]. Briefly, the Color Deconvolution tool was employed in each selected area (esophagus stratified squamous epithelium) to separate the DAB signal channels from the hematoxylin counterstaining. The final data were calculated as the mean optical density of the selected area measured as average optical density.

### Statistical analysis

Based on the normality test results, quantitative data between two groups were compared using the *t*-test or Mann–Whitney *U* test, and for more than two groups, ANOVA with Tukey's post-hoc test or Kruskal–Wallis with Dunn's post-hoc test was used. Qualitative data were compared using Fisher's exact test. Diagnostic performance of hub genes was measured by generating receiver operating characteristic curves (ROC) and choosing the optimal cut-off point afterward. *P* < 0.05 were regarded statistically significant.

## Results

### Subjects’ characteristics

Clinical characteristics of all 62 enrolled pediatric subjects are summarized in Table 1. No significant difference was found in clinical symptoms, atopic comorbidities, endoscopic results, histological eosinophil counts, and peripheral blood tests between the PPI-NR and PPI-R groups. The clinical characteristics of the subset of patients who underwent transcriptome analysis are provided in Supplementary Table S1, showing no significant differences between PPI-R and PPI-NR groups within this cohort.

**Table 1:** Clinical characteristics of all subjects (*n* = 62).

	Control group (*n* = 14)	PPI-NR group (*n* = 28)	PPI-R group (*n* = 21)
Male sex, no. (%)	8 (57)	23 (82)	15 (71)
Age (y), mean ± SD	10.79 ± 4.45	8.92 ± 5.69	8.67 ± 5.14
Symptoms, no. (%)	14 (100)	21 (95)	13 (81)
Dysphagia	7 (50)	7 (32)	3 (19)
Food impaction	1 (7)	9 (41)	3 (19)
Heartburn	1 (7)	4 (18)	3 (19)
Chest pain	0 (0)	1 (5)	3 (19)
Abdominal pain	4 (29)	6 (27)	4 (25)
Nausea	1 (7)	1 (5)	3 (19)
Vomiting	3 (21)	5 (23)	8 (50)
Atopic comorbidities, no. (%)	0 (0)^a^****^b^***	14 (64)	9 (56)
Allergic rhinitis/sinusitis	0 (0)	6 (27)	2 (12)
Eczema	0 (0)	0 (0)	0 (0)
Asthma	0 (0)	3 (14)	1 (6)
Atopic dermatitis	0 (0)	1 (5)	1 (6)
Food allergy	0 (0)^a^**^b^**	10 (45)	8 (50)
Endoscopic appearance, no. (%)	5 (36)^a^***^b^*	25 (93)	16 (76)
Rings	0 (0)^a^**	13 (48)	6 (29)
Stricture	0 (0)	4 (15)	1 (5)
Furrows	5 (36)^a^***	25 (93)	15 (71)
Edema	4 (29)^a^**^b^*	22 (81)	14 (67)
Exudates	2 (14)^a^***^b^*	19 (70)	12 (57)
Histology eos/HPF, mean ± SD	0.00 ± 0.00^a^***^b^***	43.20 ± 25.78	29.05 ± 18.19

*P < 0.05; **P < 0.01; ***P < 0.001; ****P < 0.0001. ^a^Comparison between PPI-NR and controls; ^b^Comparison between PPI-R and controls; Eos/HPF: eosinophils per high-power field. Note: Clinical data were partially missing in retrospectively included patients from external centers. In the PPI-NR group, data on clinical symptoms and atopic comorbidities were unavailable for six patients, and endoscopic findings were unavailable for one patient. In the PPI-R group, data on clinical symptoms and atopic comorbidities were unavailable for five patients, and endoscopic findings were unavailable for one patient.

### Esophageal transcriptome comparison between PPI-NR and PPI-R

**Figure 2:**
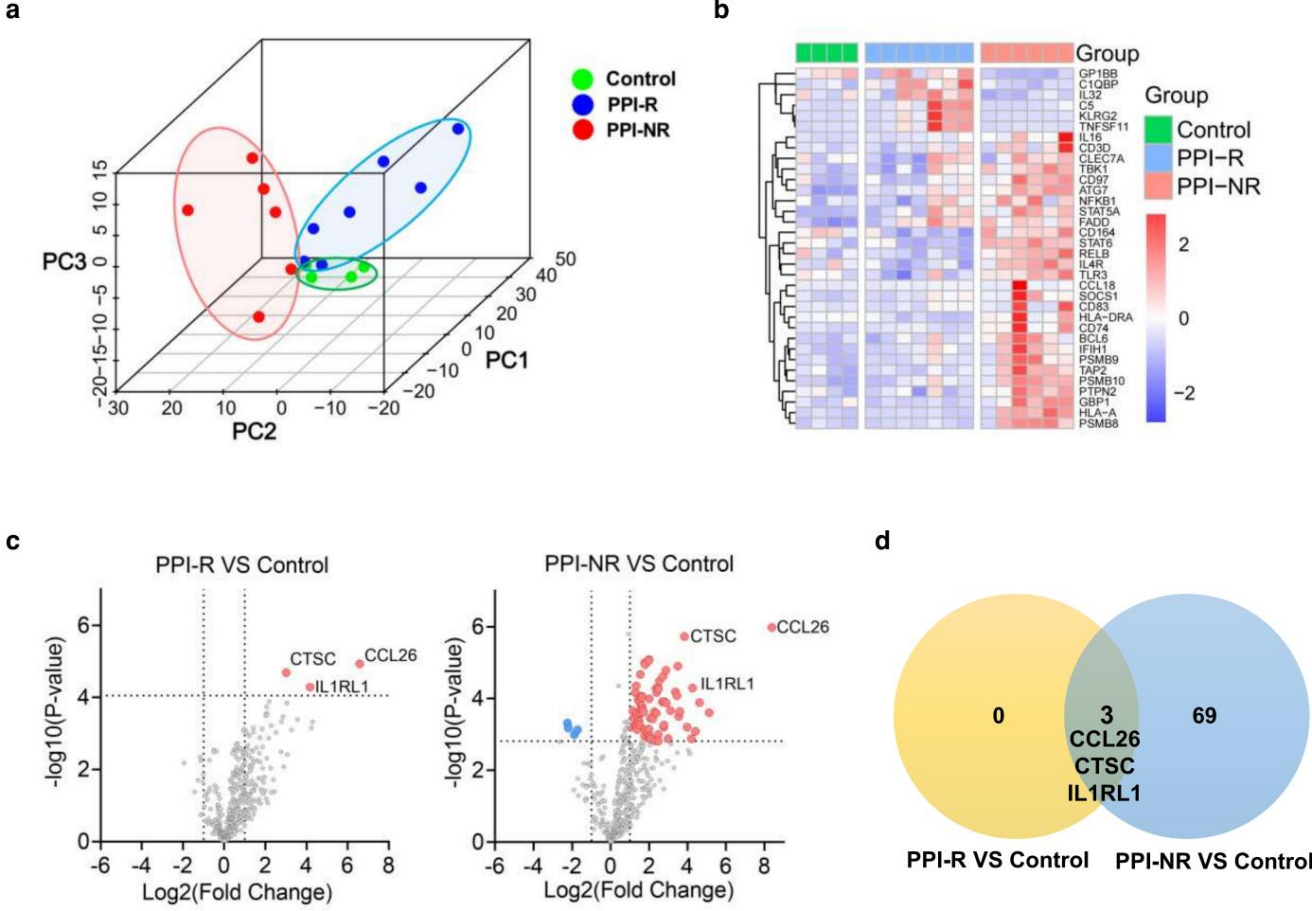
(a) 3D scatterplots of principal component analysis showing a clear separation between PPI-NR and PPI-R clusters. (b) Heatmap displays the 34 most representative genes (adj. *P* < 0.1) within the PPI-R and PPI-NR cohorts. Red indicates higher expression and blue represents lower expression. (c) Volcano plots depict differentially expressed RNAs (Log FC >1, adj. *P* < 0.05). (d) VENN diagram of differentially expressed genes that are exclusive or common to PPI-NR and PPI-R groups.

For an initial data quality assessment, we performed PCA to visualize the overall effect of experimental covariates across samples. PCA comprising all 594 genes revealed three distinguishable groups, indicating a clear separation between PPI-NR and PPI-R clusters. The variance of principal components 1, 2, and 3 accounted for 46.76%, 18.96%, and 7.55% of the total variance (Fig. 2a). To further validate these findings, we performed differential expression analysis using the statistically robust advanced analysis module in nSolver Analysis Software. The heatmap generated of the representative genes with the highest significance in differentiation of esophageal immunology transcriptome between PPI-NR and PPI-R patients showed a strict polarization between the PPI-NR and PPI-R (adj. *P* < 0.1; Fig. 2b). When comparing gene expression patterns in esophageal tissues across all groups, we found three genes were differentially expressed in the PPI-R group while 72 genes were differentially expressed in the PPI-NR group compared to controls (Fig. 2c). Three characteristic EoE signature genes CCL26, CTSC, and IL1RL1 were commonly increased in both PPI-R and PPI-NR groups, while further 69 DEGs were PPI-NR-specific (Fig. 2c, d). This demonstrated that the immunological transcriptomic profile partially differs between PPI-NR and PPI-R. While both groups share elevated gene expressions of CCL26, CTSC, and IL1RL1 that distinguish the common cohort comprising all EoE patients from healthy subjects, 69 genes were indicative of the EoE disease, especially in PPI-NR patients (Fig. 2d).

### Identification of DEGs between PPI-R and PPI-NR and functional enrichment analyses

**Figure 3:**
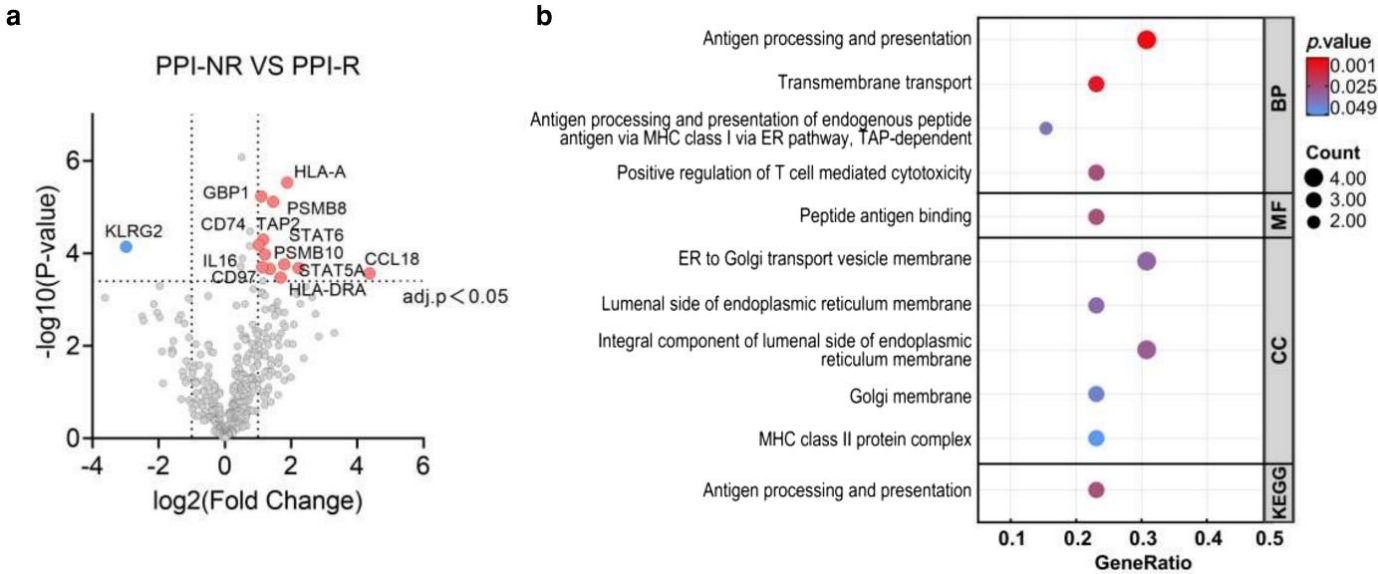
(a) Volcano plots depicting differentially expressed genes between PPI-NR and PPI-R groups (|Log FC| > 1, adj. *P* < 0.05). (b) Bubble chart shows enrichment of differentially expressed genes in GO terms and KEGG signaling pathways.

Next, a differential expression analysis of esophageal transcriptomes was performed between the PPI-R and PPI-NR groups. This analysis identified 13 DEGs (Log2FC >1, adj. *P* < 0.05), with 12 genes upregulated and 1 gene downregulated in the PPI-NR group compared to the PPI-R group (Fig. 3a). GO and KEGG enrichment analysis of these13 DEGs was conducted to further delineate the dysregulated pathway and biological process between PPI-NR and PPI-R groups (Fig. 3b and Supplementary Table S2). Both GO and KEGG pathway enrichment analyses revealed significant enrichment of DEGs in the ‘Antigen processing and presentation’ pathway. Additionally, GO enrichment analysis demonstrated that most terms enriched among the DEGs are also related to ‘antigen processing and presentation’, including terms like TAP-dependent and peptide antigen. These findings highlight a dysregulation of antigen processing and presentation in PPI-NR patients.

### Protein–protein interaction network analysis and hub gene selection

**Figure 4:**
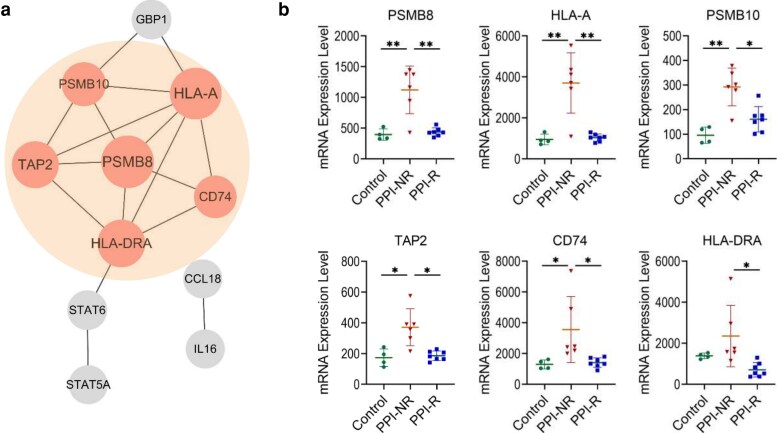
(a) The protein–protein interaction network of differentially expressed genes identified between PPI-NR and PPI-R patients, the significant gene clusters identified by MCODE are noted with orange colors. The node size reflects the connectivity degree (MCC value), with larger diameter indicating higher degrees. (b) mRNA expression of the top six hub genes among all groups (normalized data). * adjusted *P* < 0.05; ** adjusted *P* < 0.01.

The protein–protein interaction network of DEGs was generated by STRING and Cytoscape (Fig. 4a). The local clustering coefficient was calculated as 0.633, with a significant protein–protein interaction enrichment (*P* = 5.77e−15). The most significant gene cluster in the network was identified by MCODE (Fig. 4a, Supplementary Table S3). Simultaneously, the top six genes (PSMB8, HLA-A, PSMB10, TAP2, CD74, and HLA-DR) with the highest connectivity within the network were recognized by the MCC method of Cytohubba (Fig. 4a, Table 2). These six genes also belonged to the most significant gene clusters identified by MCODE. According to these findings, we suggest that these six genes represent hub genes in the protein–protein interaction network, and play essential roles in differentiating pathological processes between PPI-NR and PPI-R groups with the potential to serve as reliable predictors. Furthermore, differential expression analysis revealed that the mRNA expression levels of these six hub genes were all significantly upregulated in the PPI-NR group compared to the PPI-R group (adj. *P* < 0.05, Fig. 4b).

**Table 2:** The top six hub genes identified by Cytohubba.

Gene symbol	Description	MCC	Node degree	Log2FC (PPI-NR vs PPI-R)
PSMB8	Proteasome subunit beta type-8	20	6	1.46
HLA-A	HLA class I histocompatibility antigen, A-3 alpha chain	18	5	1.88
HLA-DRA	HLA class II histocompatibility antigen, DR alpha chain	13	5	1.79
TAP2	Antigen peptide transporter 2	12	4	1.16
PSMB10	Proteasome subunit beta type-10	8	4	1.13
CD74	HLA class II histocompatibility antigen gamma chain	6	3	1.36

### PSMB8 expression predicts PPI-responsiveness

We further evaluated the top hub gene PSMB8 and its protein expression levels in the esophagus epithelium in an extended cohort. This cohort comprised 28 PPI-NR, 20 PPI-R patients, and 14 controls (19 PPI-NR, 10 PPI-R patients, and 14 controls prospectively collected from Erlangen Hospital, along with 9 PPI-NR and 10 PPI-R patients retrospectively collected from four additional institutions)

**Figure 5:**
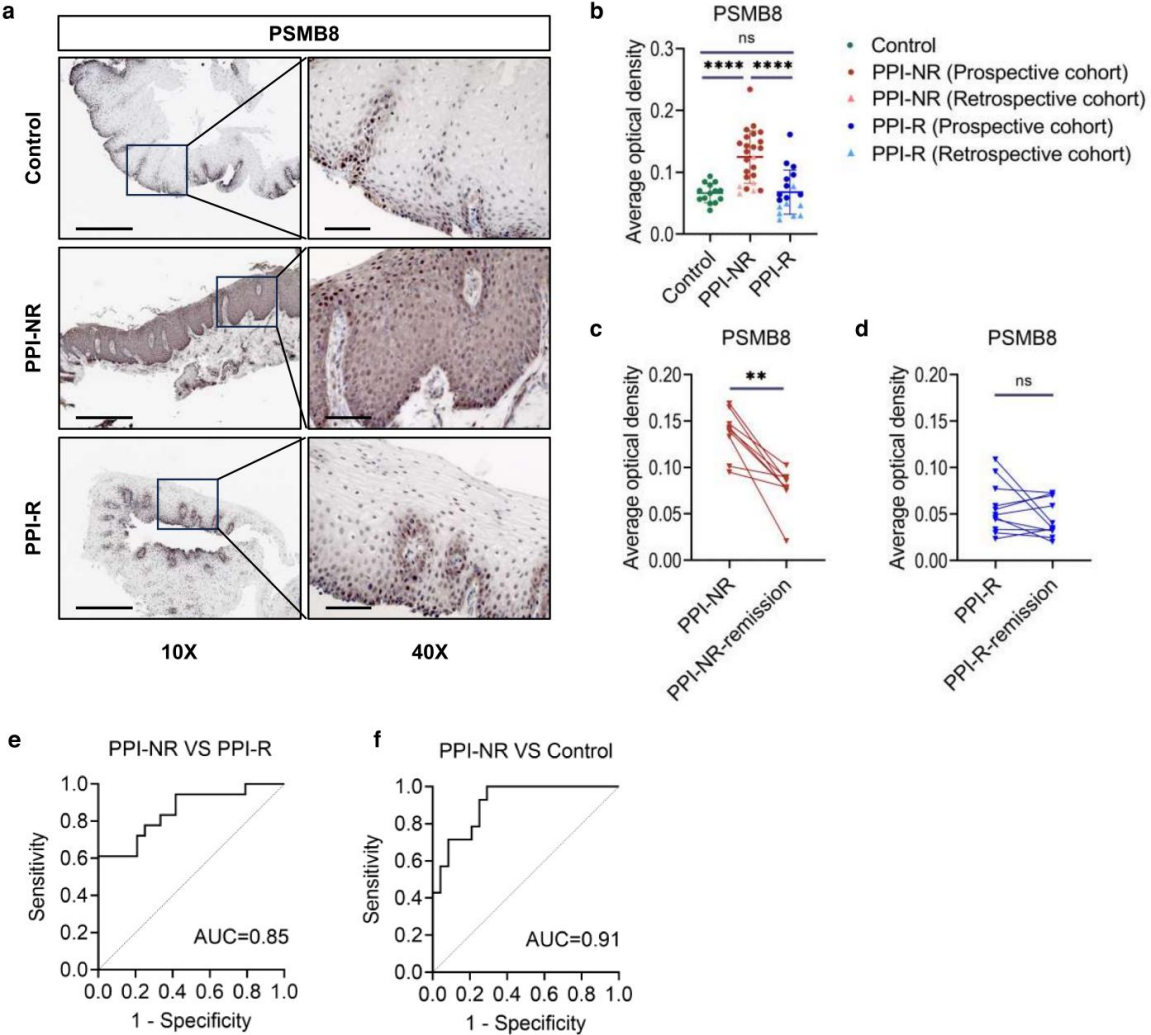
(a) Esophageal tissues were evaluated by immunohistochemistry for PSMB8 (10× objective, scale bars: 5 μm; 40× objective, scale bars: 1 μm). (b) Semi-quantitative assessment of PSMB8 (prospective cohort: PPI-NR *n* = 19; PPI-R *n* = 10, control *n* = 14; retrospective cohort: PPI-NR *n* = 5; PPI-R *n* = 8). (c) Semiquantitative assessment of PSMB8 between the PPI-NR and PPI-NR-remission group. (d) Semiquantitative assessment of PSMB8 between the PPI-R and PPI-R-remission group. (e) Receiver operating characteristic curves of PSMB8 protein expression level in esophagus samples to predict PPI responsiveness. **P < 0.01, ****P < 0.0001.

IHC demonstrated that PSMB8 was expressed in almost the entire layer of the esophageal epithelium of PPI-NR patients, contrasting with its expression restricted to the basal layer in PPI-R and control groups (Fig. 5a). The semi-quantitative analysis further demonstrated significantly higher PSMB8 expression in PPI-NR patients compared to the PPI-R group (PSMB8: *P* < 0.0001; Fig. 5b). Moreover, IHC staining of patients achieving remission showed a significant decrease in PSMB8 level in PPI-NR patients (PSMB8: *P* < 0.01; Fig. 5c), with no change in the PPI-R patients (Fig. 5d).

To evaluate the predictive value of PSMB8 IHC staining for the diagnosis of EoE and PPI responsiveness in esophageal tissue, ROC curves were performed based on the semi-quantitative IHC scores from all subjects. PSMB8 showed high diagnostic significance for PPI responsiveness (Fig. 5e, f; Tables 3 and 4).

**Table 3:** AUC calculations of IHC results for PPI-NR, PPI-R, and controls.

	PPI-NR vs controls				PPI-NR vs PPI-R			
	AUC	95% CI		*P*	AUC	95% CI		*P*
PSMB8	0.91	0.82	1.00	<0.0001	0.85	0.74	0.97	0.0001

**Table 4:** PSMB8 cut-off point.

	PPI-NR vs controls			PPI-NR vs PPI-R		
	Cut-off point	Sensitivity	Specificity	Cut-off point	Sensitivity	Specificity
PSMB8	<0.09	1.00	0.71	<0.06	0.61	1.00

Furthermore, we performed ELISA assays on serum samples from PPI-NR and PPI-R-patients to assess the possibility of cellular release of PSMB8. However, no significant differences in PSMB8 protein expression in serum were observed (Supplementary Figure S1).

## Discussion

Proton-pump inhibitors represent an effective therapy option for EoE. However, about 25–50% of EoE patients show a primary non-response to a PPI therapy [26, 27]. Previous transcriptome studies in adults demonstrated barely any differences between PPI-NR and PPI-R, indicating these two groups share the same spectrum of a Th_2_-mediated disease process [10–12]. In this study, we have identified for the first time a cluster of 13 genes differentially expressed between the PPI-R and PPI-NR groups in pediatric subjects. The enrichment analysis based on these DEGs indicated the association of PPI-refractory with the antigen processing and presentation pathway. Our results further demonstrate that hub gene PSMB8 may represent predictive biomarkers indicating PPI responsiveness in EoE patients.

Indeed, we can confirm a partially consistent transcriptional profile between PPI-responders and non-responders according to three genes (CCL26, IL1RL1, and CTSC) that are similarly upregulated compared to the controls (Fig. 2d). Especially, CCL26 and IL1RL1 have been extensively studied in the pathophysiology of EoE [28, 29]. IL1RL1 (ST2) is the receptor of IL-33, the genetic deletion of IL1RL1 has been shown to prevent inflammation in an OVA-induced EoE mouse model. Moreover, the upregulated IL-33-ST2 axis promotes Th2-mediated inflammation [30, 31], contributing to eosinophil chemotaxis by inducing CCL26 (eotaxin-3) expression [32, 33], a signature gene of EoE. Cathepsin C is an important lysosomal cysteine protease that is involved in the maturation of proinflammatory granule-associated serine proteases and the immune regulation associated with polymorphonuclear neutrophils [34]. Despite limited reports on CTSC's role in EoE pathogenesis, a transcriptomic analysis linking EoE and atopic dermatitis is in line with our findings as it revealed high CTSC mRNA expression in EoE, atopic dermatitis, and allergic airway diseases [35]. However, it is worth noting that the PCA showed clear separation between PPI-NR and PPI-R associated clusters (Fig. 2a). Compared to controls, three genes were dysregulated in the PPI-responders while 72 genes were dysregulated in PPI-non-responders (Fig. 2c). This more profound dysregulation of immunological gene expression observed in PPI-non-responders raises the hypothesis that PPI-responsiveness may represent an early stage of inflammation in EoE development. This profound dysregulation in the PPI-NR cohort might explain the PPI therapy refractoriness.

Additionally, 13 immune-related genes showed significant differences between the two groups (Fig. 2a). These findings differ from previous transcriptomic studies of PPI responses [10–12], possibly attributed to variations in sample selection strategy. Specifically, our study exclusively employed proximal esophageal specimens from therapy-naive pediatric subjects, whereas earlier studies analyzed distal esophageal samples from adults. Recent research suggests age-related differences in the EoE transcriptome, resulting in significant differences in cell composition and pathway regulation between adult and pediatric EoE patients [36]. Furthermore, gastroesophageal reflux may alter transcriptional profiles in distal tissue, confirming our sample selection strategy to avoid such confounding effects. To further validate the representativeness of proximal biopsies, we compared matched proximal and distal segment biopsies within our cohort. Our findings demonstrated that both immune-related gene expression profiles and eosinophilic infiltration were remarkably consistent across these locations (Supplementary Figures S2 and S3), indicating that the proximal tissue effectively captures the global esophageal molecular landscape in pediatric EoE patients. In particular, results from several other groups may support our hypothesis of a ‘developmental inflammatory march’, for instance. Gutiérrez and Ting reported lower eosinophils/HPF in PPI-R patients compared to PPI-non-responders [12, 37]. Sayej and Molina also revealed that patients with more severe histological results had a lower PPI response rate [38, 39].

Functional enrichment analysis based on the 13 DEGs revealed that the most significant GO terms and KEGG pathways are both related to ‘Antigen processing and presentation’. Antigen presentation is crucial for initiating allergic immune response in EoE [40]. Nahoko et al. found lower filaggrin expression in PPI-NR patients’ esophageal epithelium than PPI-R patients, which may lead to increased influx of allergen antigens into the esophageal epithelium [41]. Furthermore, other studies found elevated levels of antigen presentation-related proteins in the esophageal proteomes of PPI-NR patients [42]. Notably, our findings are highly consistent with a recent transcriptomic study by Chakraborty et al. [43], which similarly observed an enrichment of antigen presentation pathways in patients who did not respond to PPI therapy. The reproducibility of this signature across different independent cohorts underscores the critical role of dysregulated antigen handling in PPI refractoriness. Our results further suggest that PPI-NR patients may have a more vulnerable esophageal barrier, raising the probability of an increased antigen exposure and thereby exacerbating EoE, as previously described [44–46]. While these results require further investigation, our enrichment analysis highlights differences in antigen processing and presentation functions between PPI-NR and PPI-R patients. Thus, we speculate that a severe esophageal barrier impairment in PPI-NR patients may lead to enhanced antigen processing pathways involving relevant hub genes such as PSMB8 leading to a more severe stage of the inflammatory response that may cause PPI-refractoriness. In contrast, PPI-R patients might exhibit milder epithelial dysfunction and inflammation, sufficient to respond to PPI therapy.

In this study, transcriptomic analysis identified PSMB8 as a hub gene between PPI-NR and PPI-R patients, subsequent IHC staining confirmed its markedly higher expression in the PPI-NR group on the protein level, demonstrating strong predictive power for distinguishing between the two groups (Fig. 5e). Proteasome 20S subunit beta 8 (PSMB8) is a proteolytically active subunit of immunoproteasome induced under inflammatory conditions [47]. An essential function of PSMB8 is to process intracellular proteins into antigenic peptides [48]. Yet, emerging evidence suggests that immunoproteasomes have a more essential role in adaptive immune responses than antigen processing effects, including the regulation of transcription factors [49]. Additionally, it has roles in inflammatory responses, including those seen in autoimmune diseases and infections. Its role in allergy (type 2 immune responses) is less clear and currently under investigation [50].

Of importance is that the knockout of immunoproteasome subunits reduces key transcription factors for type 2 inflammation, such as STAT6 [51]. Notably, PPI such as omeprazole was found to reduce the Eotaxin-3 mRNA transcription by inhibiting the STAT6 pathway [7, 8], hence demonstrating a direct immunological effect in the therapy of EoE. In this respect, it is a noteworthy finding that our transcriptome analysis showed significantly higher STAT6-mRNA levels in the PPI-NR compared to the PPI-R group (adj. *P* < 0.05, Fig. 3a).

Although the study is characterized by a small sample size, with gene expression profiling conducted on 18 samples, we were successful in demonstrating the generalizability and robustness of our findings as the identified hub genes were validated across all 62 participants (43 from our center and 19 participants from four other German institutions)—clearly representing a strength of this study. Despite these efforts, a larger-scale, multicenter registry is currently established for further validation in Germany. To address a potential selection bias between the analytic cohort (subjects included in gene expression profiling) and the non-analytic cohort (subjects not included in gene expression profiling), we compared the clinical characteristics of the analytic and non-analytic cohorts within the control, PPI-NR, and PPI-R groups. No significant differences were found in the control and PPI-NR groups (Supplementary Tables S4 and S5). However, in the PPI-R group, there were more males in the non-analytic cohort compared to the analytic cohort (92% vs. 43%, *P* = 0.03, Supplementary Table S6)—a well-known observation described in previous reports and EoE registries [52].

However, our study has several technical limitations that warrant consideration. First, the use of the nCounter^®^ Human Immunology v2 Panel restricted our analysis to 594 pre-selected immune-related genes. While this targeted digital profiling provides high-precision data with superior reproducibility for esophageal inflammatory signatures, it does not constitute an exhaustive transcriptomic survey. Consequently, potential non-immunologic drivers of PPI-non-responsiveness, such as those related to epithelial barrier integrity or esophageal remodeling, might have been overlooked. Future studies utilizing unbiased whole-transcriptome RNA sequencing (RNA-seq) may provide a more comprehensive landscape of the molecular mechanisms underlying therapy resistance in EoE.

## Conclusion

Taken together, the difference in PSMB8 expression between PPI-responders and non-responders provides new immunological insights for the initiation phase of EoE pathophysiology. Furthermore, integrating immunohistochemical staining of PSMB8 have—due to the test sensitivity and specificity—the potential to improve the physician’s treatment decision at initial EoE diagnosis to implement an individualized therapy strategy.

## Supplementary Material

uxag012_Supplementary_Data

## Data Availability

The data that support the findings of this study are available from the corresponding author upon reasonable request.

## Clinical trial registration

The study was officially registered at DRKS #00014688.
