# Supplementary material for: PSMB8 stratifies therapy response in eosinophilic esophagitis
Source: Clin Exp Immunol. 2026 Apr 7;220(1):uxag012. doi: 10.1093/cei/uxag012 (PMC13062531; doi:10.1093/cei/uxag012)
Supplement: uxag012_Supplementary_Data [file uxag012_supplementary_data.docx]

## Supplementary Tables

### Table S1

| **Table S1. Clinical characteristics of subjects undergoing transcriptome analysis** | | | |
| --- | --- | --- | --- |
|  | **Control group**  **(n=4)** | **PPI -NR group**  **(n=6)** | **PPI-R group**  **(n=7)** |
| Male sex, no. (%) | 2 (50) | 4(67) | 3(43) |
| Age (y), mean ± SD | 10.77 ±5.97 | 9.58±5.22 | 7.49±4.81 |
| Symptoms, no. (%) | 4 (100) | 6(100) | 6(86) |
| Dysphagia | 1 (25) | 3(50) | 2(29) |
| Food impaction | 0 (0) | 3(50) | 1(14) |
| Heartburn | 0 (0) | 1(17) | 0(0) |
| Chest pain | 0 (0) | 0 (0) | 0 (0) |
| Abdominal pain | 2 (50) | 3(50) | 0(0) |
| Nausea | 0 (0) | 1(17) | 1(14) |
| Vomiting | 2 (50) | 2(33) | 5(71) |
| Atopic comorbidities, no. (%) | 0 (0) †* | 5(83) †* | 3(42) |
| Allergic rhinitis/sinusitis | 0 (0) | 1(17) | 2(29) |
| Eczema | 0 (0) | 0 (0) | 0 (0) |
| Asthma | 0 (0) | 0 (0) | 1(14) |
| Atopic dermatitis | 0 (0) | 0 (0) | 0 (0) |
| Food allergy | 0 (0) †* | 5(83) †* | 3(42) |
| Endoscopic appearance, no. (%) | 0 (0) †**‡* | 6(100) †** | 6(86) ‡* |
| Rings | 0 (0) | 4(67) | 2(29) |
| Stricture | 0 (0) | 0 (0) | 0 (0) |
| Furrows | 0 (0) †**‡* | 6(100) †** | 6(86) ‡* |
| Edema | 0 (0) †** | 6(100) †** | 5(71) |
| Exudates | 0 (0) †* | 5(83) †* | 3(43) |
| Histology Eos/ HPF, mean ± SD | 0.00 ± 0.00†***‡** | 46.83±17.74†*** | 31.67±15.58‡** |

**p*<0.05; ***p*<0.01; ****p*<0.001; † Comparison between PPI-NR and Control; ‡ Comparison between PPI-R and Control; §Comparison between PPI-NR and PPI-R; Eos/ HPF:  eosinophil per high-power field;

### Table S2

| **Table S2. The significantly enriched GO term and KEGG pathways by DEGs involving PPI-R with PPI-NR diseases.** | | | | | | |
| --- | --- | --- | --- | --- | --- | --- |
|  | **ID** | **Description** | **Count** | **Gene Ratio (%)** | **p value** | **Gene ID** |
| **BP** | GO:0019882 | antigen processing and presentation | 4 | 30.77 | 0.004 | CD74, HLA-DRA, HLA-A, PSMB8 |
|  | GO:0055085 | transmembrane transport | 3 | 23.08 | 0.008 | TAP2, PSMB8, PSMB10 |
|  | GO:0002485 | antigen processing and presentation of endogenous peptide antigen via MHC class I via ER pathway, TAP-dependent | 2 | 23.08 | 0.037 | TAP2, HLA-A |
|  | GO:0001916 | positive regulation of T cell mediated cytotoxicity | 3 | 23.08 | 0.041 | TAP2, HLA-DRA, HLA-A |
| **MF** | GO:0042605 | peptide antigen binding | 3 | 23.08 | 0.048 | TAP2, HLA-DRA, HLA-A |
| **CC** | GO:0012507 | ER to Golgi transport vesicle membrane | 3 | 30.77 | 0.026 | CD74, HLA-DRA, HLA-A |
|  | GO:0098553 | lumenal side of endoplasmic reticulum membrane | 3 | 23.08 | 0.026 | CD74, HLA-DRA, HLA-A |
|  | GO:0071556 | integral component of lumenal side of endoplasmic reticulum membrane | 3 | 15.38 | 0.026 | CD74, HLA-DRA, HLA-A |
|  | GO:0000139 | Golgi membrane | 4 | 23.08 | 0.033 | CD74, HLA-DRA, HLA-A, GBP1 |
|  | GO:0042613 | MHC class II protein complex | 3 | 23.08 | 0.034 | CD74, HLA-DRA, HLA-A |
| **KEGG** | hsa04612 | Antigen processing and presentation | 4 | 30.77 | 0.030 | CD74, TAP2, HLA-DRA, HLA-A |

GO: Gene Ontology; KEGG: Kyoto Encyclopaedia of Genes and Genomes; DEGs: differentially expressed genes; PPI-NR: proton pump inhibitor-non-responders; PPI-R: proton pump inhibitor-responders; BP: Biological Process; MF: Molecular Function; CC: Cell Components.

### Table S3

| **Table S3.** The gene clusters identified by MCODE | | | | |
| --- | --- | --- | --- | --- |
| **Cluster** | **Score (Density*#Nodes)** | **Nodes** | **Edges** | **Node IDs** |
| 1 | e | 6 | 12 | PSMB8, HLA-A, HLA-DRA, TAP2, PSMB10, CD74 |

### Table S4

| **Table S4. Comparison of Clinical Characteristics Between Transcriptome Analytic and Non-Analytic Cohorts in Control Group** | | | |
| --- | --- | --- | --- |
|  | **Analysis cohort**  **(n=4)** | **Non-analysis cohort**  **(n=10)** | **p-value** |
| Male sex, no. (%) | 2 (50) | 6(60) | >0.99 |
| Age (y), mean ± SD | 10.77 ±5.97 | 10.00±4.67 | 0.57 |
| Endoscopic appearance, no. (%) | 0 (0) | 5(50) | 0.22 |
| Rings | 0 (0) | 0 (0) | >0.99 |
| Stricture | 0 (0) | 0 (0) | >0.99 |
| Furrows | 0 (0) | 5(50) | 0.22 |
| Edema | 0 (0) | 4(40) | 0.25 |
| Exudates | 0 (0) | 2(20) | >0.99 |
| Histology eos/ HPF, mean ± SD | 0.00 ± 0.00 | 0.00 ± 0.00 | >0.99 |

Analytic cohort: healthy control subjects included in gene expression profiling; Non-analytic cohort: healthy control subjects not included in gene expression profiling.

### Table S5

| **Table S5. Comparison of Clinical Characteristics Between Transcriptome Analytic and Non-Analytic Cohorts in PPI-NR Group** | | | |
| --- | --- | --- | --- |
|  | **Analysis cohort**  **(n=6)** | **Non-analysis cohort**  **(n=22)** | **p-value** |
| Male sex, no. (%) | 4(67) | 19(86) | 0.29 |
| Age (y), mean ± SD | 9.58±5.22 | 8.75±5.91 | 0.84 |
| Endoscopic appearance, no. (%) | 6(100) | 21(100) | >0.99 |
| Rings | 4(67) | 9(43) | 0.38 |
| Stricture | 0(0) | 4(19) | 0.55 |
| Furrows | 6(100) | 19(90) | >0.99 |
| Edema | 6(100) | 16(76) | 0.56 |
| Exudates | 5(83) | 14(67) | 0.63 |
| Histology eos/ HPF, mean ± SD | 46.83±17.74 | 43.11±26.97 | 0.5 |

Analytic cohort: healthy control subjects included in gene expression profiling; Non-analytic cohort: healthy control subjects not included in gene expression profiling. Endoscopic appearance results were unavailable for one patient in the PPI-NR Non-analytic cohort.

### Table S6

| **Table S6. Comparison of Clinical Characteristics Between Transcriptome Analytic and Non-Analytic Cohorts in PPI-R Group** | | | |
| --- | --- | --- | --- |
|  | **Analysis cohort**  **(n=7)** | **Non-analysis cohort**  **(n=13)** | **p-value** |
| Male sex, no. (%) | 3(43) | 12(92) | 0.03 |
| Age (y), mean ± SD | 7.49±4.81 | 9.33±5.42 | 0.43 |
| Endoscopic appearance, no. (%) | 6(86) | 10(83) | >0.99 |
| Rings | 2(29) | 4(33) | >0.99 |
| Stricture | 0 (0) | 0(0) | >0.99 |
| Furrows | 6(86) | 9(75) | >0.99 |
| Edema | 5(71) | 9(75) | >0.99 |
| Exudates | 3(43) | 9(75) | 0.33 |
| Histology eos/ HPF, mean ± SD | 31.67±15.58 | 30.64±19.81 | 0.59 |

Analytic cohort: healthy control subjects included in gene expression profiling; Non-analytic cohort: healthy control subjects not included in gene expression profiling. Endoscopic appearance results were unavailable for one patient in the PPI-R Non-analytic cohort.

# Supplementary Figures

### Figure S1


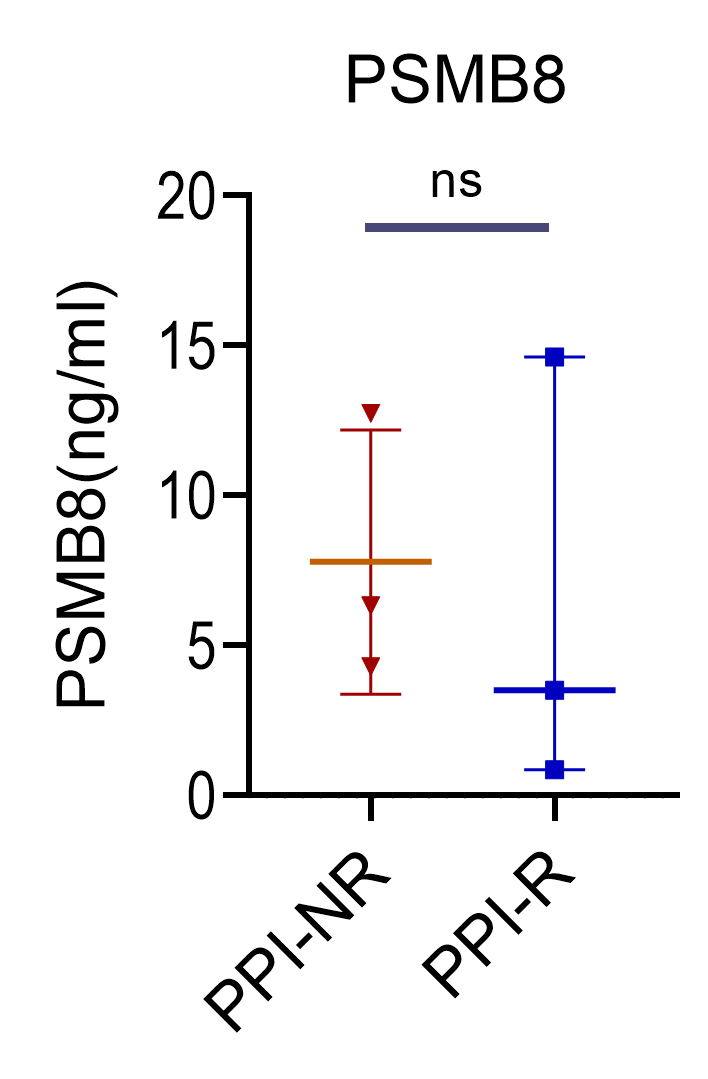


**Figure S1**. PSMB8 concentrations in serum samples from patients with PPI-NR and PPI-R (PPI-NR n=3; PPI-R n=3). PPI-NR: proton pump inhibitor-non-responders; PPI-R: proton pump inhibitor-responders.

### Figure S2


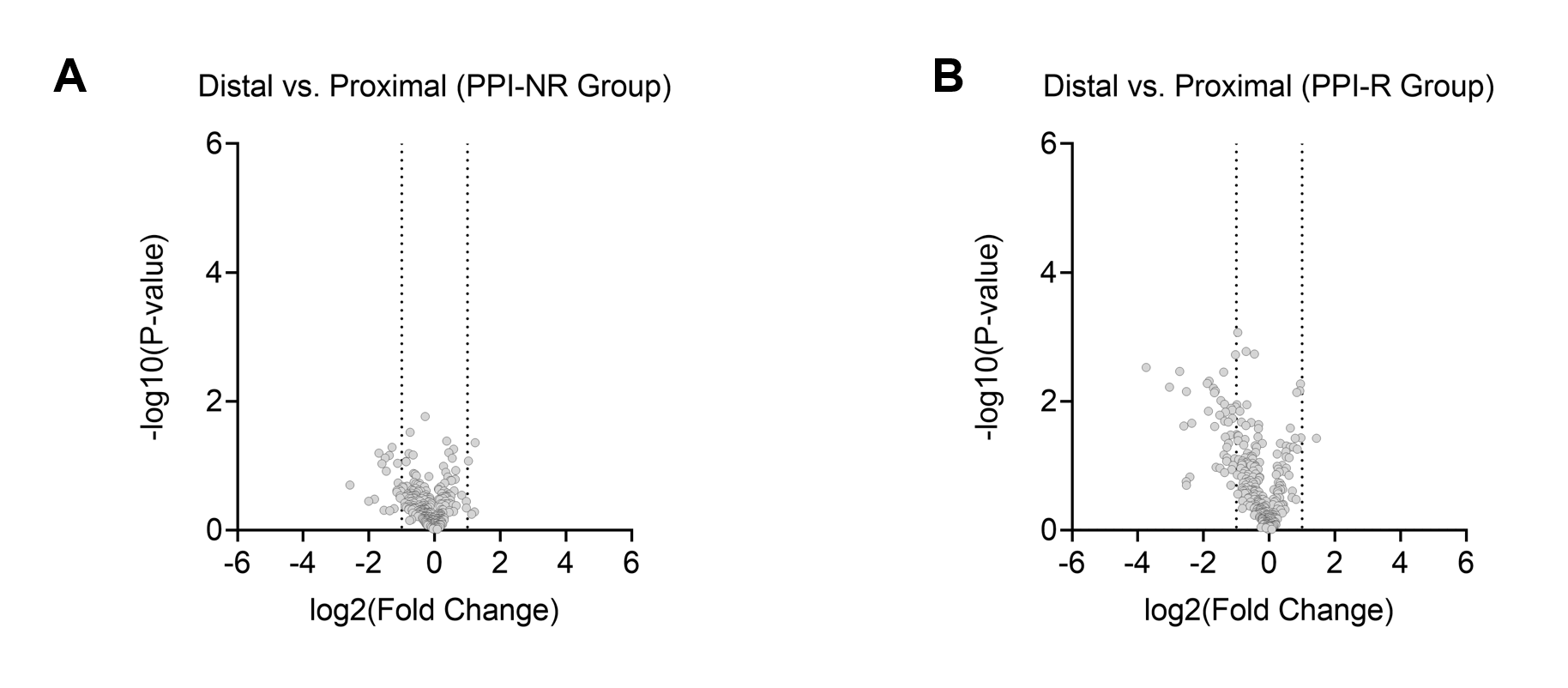


**Figure S2.** Comparison of immune-related gene expression profiles between matched proximal and distal esophageal segments. Volcano plots show that no differentially expressed genes were identified between proximal and distal biopsies in the PPI-NR group **(A)** and the PPI-R group **(B)** using thresholds of |log2FC| > 1 and adjusted p < 0.05.

### Figure S3


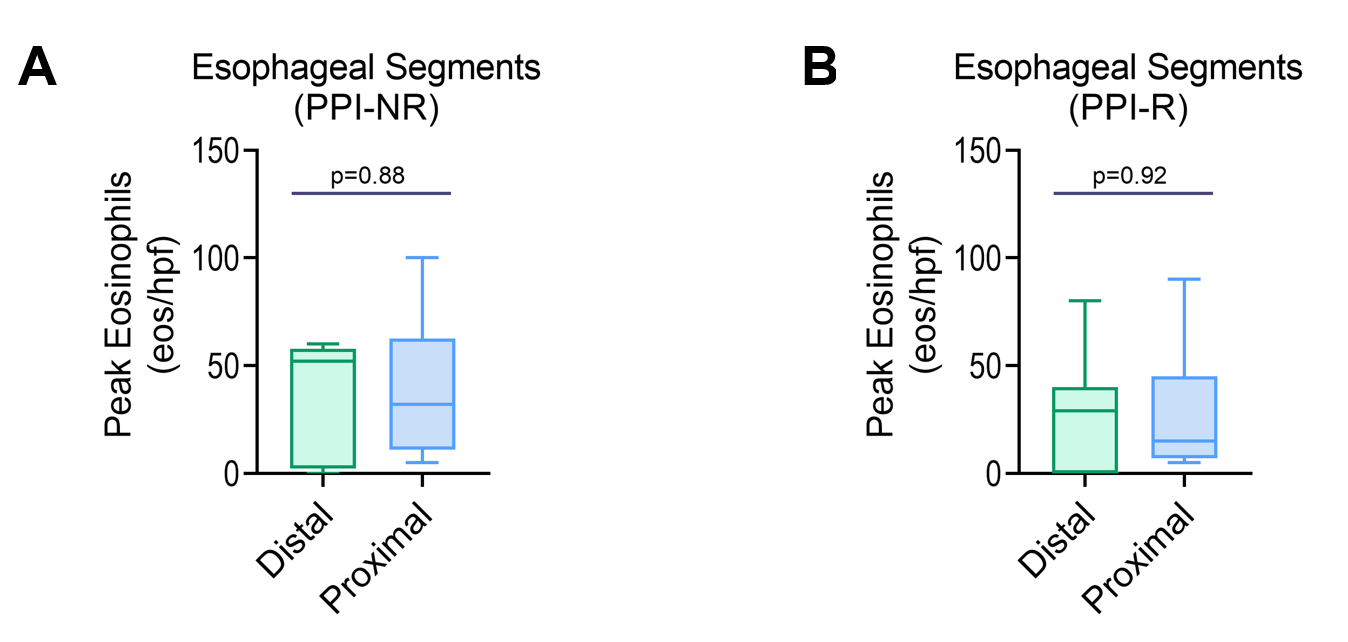


**Figure S3.** Comparison of eosinophilic infiltration between matched proximal and distal esophageal segments. Boxplots indicate no significant difference in peak eosinophil counts (eos/hpf) between proximal and distal biopsies in the PPI-NR group (A) and the PPI-R group (B).
